# Application of New Nanoparticle Structures as Catalysts

**DOI:** 10.3390/nano10091686

**Published:** 2020-08-27

**Authors:** Antonio Guerrero Ruiz, Inmaculada Rodríguez-Ramos

**Affiliations:** 1 Department of Inorganic and Technical Chemistry, Facultad de Ciencias UNED, 28040 Madrid, Spain; 2 Instituto de Catálisis y Petroleoquímica, CSIC, Campus Cantoblanco, 28049 Madrid, Spain

Nanocatalysts, more precisely solids nanomaterials with catalytic properties to be used as heterogeneous catalysts, are an extended and very diverse group of nanostructured materials representing, at present, an active area of research with application in many catalyzed processes. Therefore, this research area not only can lead to significant advances for their potential technological applications, but also must engage a large variety of new materials. For the characterization of the studied nanocatalysts, many different techniques and experimental methods should be used to reveal both the structural and superficial properties of these materials. The scope of the Special Issue “Application of New Nanoparticle Structures as Catalysts” was to provide a non-systematic overview with current research studies in the field of developing nanocatalyts. Practically all the nanomaterials compositions presented as examples in this issue are different from each other, both in their chemical composition of the structured nanomaterials, or in relation to the catalyzed reactions where they are applied. In this way, in this Special Issue, nine selected original research paper and one comprehensive review are collected. More than 40 scientists from universities and research institutions contributed their research studies and expertise for the success of this Special Issue.

The scientific contributions are summarized in the next paragraphs.

An analysis of the recent studies concerning transition metal nitrides applied as heterogeneous catalysts is presented in the review carried out by Dr. Dongil [1]. These materials have a clear interest because they can substitute noble metals in different catalyzed processes. With these nanomaterials, numerous possibilities are opened for new structures for metal nitrides; since chemical ingredients can be combined as mono-, binary and even ternary mixtures or the addition of promoters can be accomplished during the preparation procedures. The description of the most employed synthetic methods is revisited and the application of some transition metal nitrides in different catalyzed reactions, hydrotreatments, oxidations and ammonia synthesis/decomposition is reported.

Two of the contributions of this Special Issue are related to Metal Organic Framework (MOF) nanostructures. The development of these MOF nanomaterials is, at present, one of the major subjects in fundamental research due to, among others, their potential applications as catalytic materials or as selective adsorbents. In the contribution by Zamaro et al. [2], the preparation of nanocatalysts derived from the MOF named UiO-66, when used as support for three transition elements (Cu, Co, and Fe), is described. These materials are evaluated in two CO oxidations: oxidation with air and selective oxidation in a hydrogen-rich stream. The main aim of this research is to find reaction conditions where these new nanostructures can be, for these processes, an alternative to the commercial catalysts based on expensive noble metals.

In the second contribution regarding MOFs by Ordonez et al. [3], a very important aspect when nanomaterials are proposed for a real application is emphasized. Thus, the solid material should be submitted to physic-mechanical treatments, such as grinding or pelleting, in order to transform the original powder into granules, which can be used at industrial scale. Three commercial materials ([Cu_3_(C_9_H_3_O_6_)_2_], [C_9_H_3_FeO_6_] and [C_8_H_5_AlO_5_]) were studied as methane adsorbents in a fixed bed reactor. It was concluded that all these materials suffer structural and textural modifications when subjected to pressure, and consequently their adsorption capacities are largely reduced.

Two other research papers in this issue are related with mixed transition metal oxides. In the contribution by Illán-Gómez et al. [4], the authors synthesized, characterized and tested a series of perovskites (BaFe_1−x_Cu_x_O_3_ with x = 0, 0.1, 0.3 and 0.4). The target reaction for these nanocatalysts is the soot oxidation, as method for avoiding the atmospheric contamination by exhaust gases of car engines. These perovskites catalyze both the NO_2_ oxidation of Diesel soot and, but to a lesser extent, the soot oxidation by O_2_ of Gasoline engines. The catalytic activities of these perovskites seem to be related to the amount of oxygen evolved during temperature programmed desorption experiments, which decreases when increasing the copper content.

In the case of the contribution by Cauqui et al. [5], a mixed oxide (ZrO_2_ with different loadings of Ce, Ca and Y) is used as a support of Ni nanoparticles. In this study, the synthesized nanomaterials are extensively characterized by complementary methods and techniques, and evaluated as catalysts for the aqueous-phase reforming of methanol. Focusing on the effect of the redox properties of ceria and the basicity properties induced by Ca or Y, it is revealed that the availability of Ni-metallic at the surfaces and the presence of weak basic sites, particularly derived from Ca incorporation, is the key parameter for improving the catalytic performance.

Y. Wang et al. reported on the use of the surface plasmon resonance effect, in this case in a metal nanocomposite, AuPt/N–TiO_2_, used as a photocatalyst [6]. While the Au nanoparticles were used to obtain energy from visible-light, Pt nanoparticles work as a cocatalyst, trapping the energetic electrons from the semiconductor support. With this material, the selective oxidation of benzyl alcohol under visible-light irradiation can be performed with a markedly enhanced selectivity and yield. An extensive series of irradiation experiments shed light on relevant information concerning the different steps of the photocatalytic mechanism with this material.

Fernández-Morales et al. reported a comparative study of diverse materials, in general solid catalysts with acidic surface properties, when applied to the reaction of isobutene dimerization to C8 olefins [7]. The exposed surface catalytic sites were conveniently characterized in order to interpret catalytic performances. In general, catalytic materials with a higher amount of Brønsted acid sites display improved catalytic performance, but for achieving an optimum selectivity towards C8 compounds, a combination of the nature of acidic sites and structural characteristics of the catalytic materials is required.

In the contribution by Ramirez-Barria et al. [8], an extended series of graphenic materials (doped or not with nitrogen adatoms, with different textural properties, etc.) were prepared and applied as electrocatalysts for the demanding oxygen reduction reaction. The material with nitrogen doping and with smaller grain sizes was demonstrated to be the most efficient electrocatalyst. Moreover, all nitrogen-doped graphenic materials show high tolerance to methanol poisoning and good stability.

The approach of Faroldi et al. [9] for the study of a new heterogeneous catalysts for the dehydrogenation reaction of formic acid, generating high-purity hydrogen, is also of great interest. Instead of noble metal catalysts, they prepared and characterized Ni-based catalysts supported on silica, which were doped with calcium in order to facilitate the adsorption-decomposition of the reactant. From the results of the catalytic performance (100% conversion with a 92% of selectivity to hydrogen) at a moderate reaction temperature, 160 °C, it can be concluded that these materials were very promising for this application. In fact, these results for catalytic behavior are comparable to those reported for noble metals.

Another example of a complex catalytic multicomponent material with an interesting potential application is reported by Ivars-Barcelo et al. [10]. In this case, the composite materials are based on noble metal particles (Pd or bi-metallic Ag/Pd) supported over an iron oxide (Fe_3_O_4_) with a magnetite structure. The catalytic application of these materials is the direct methane partial oxidation into value-added chemicals as formaldehyde. The presented preliminary catalytic results confirmed the potential of magnetite-supported (Ag)Pd catalysts for CH_4_ partial oxidation into formaldehyde, with incipient methane conversion starting at 200 °C, but with very high selectivity above 95%. The prepared nanocomposite materials were investigated by different physicochemical techniques, with the purpose of relating the structural and superficial properties of these nanocatalysts with their detected catalytic performances.

In conclusion, the papers collected in this Special Issue can be described as an impressionistic painting with brushstrokes of different aspects of new developments of catalytic materials. All of them include complementary features involved in the design of special nanocatalysts: preparation-treatments, intensive characterization and evaluation as catalysts in various reactions of applied interest. Although the present Special Issue can cover neither all the research of new structures used as nanocatalysts nor a complete list of application in catalyzed processes, the editors are confident that its contributions to fundamental research will offer new perspectives for the readers.
